# Scientific Developments and New Technological Trajectories in Sensor Research

**DOI:** 10.3390/s21237803

**Published:** 2021-11-24

**Authors:** Mario Coccia, Saeed Roshani, Melika Mosleh

**Affiliations:** 1 CNR—National Research Council of Italy, Collegio Carlo Alberto, Via Real Collegio, 30-10024 Moncalieri, Italy; 2 Department of Industrial Management, Faculty of Management and Accounting, Allameh Tabataba’i University, Tehran 1489684511, Iran; Roshani@atu.ac.ir; 3 Birmingham Business School, College of Social Sciences, University of Birmingham, Birmingham B15 2SQ, UK; mxm1219@alumni.bham.ac.uk

**Keywords:** sensor technology, technological trajectories, technological change, biosensors, wearable sensors, wireless sensor network, sensor network, evolution of science, scientific development

## Abstract

Scientific developments and new technological trajectories in sensors play an important role in understanding technological and social change. The goal of this study is to develop a scientometric analysis (using scientific documents and patents) to explain the evolution of sensor research and new sensor technologies that are critical to science and society. Results suggest that new directions in sensor research are driving technological trajectories of wireless sensor networks, biosensors and wearable sensors. These findings can help scholars to clarify new paths of technological change in sensors and policymakers to allocate research funds towards research fields and sensor technologies that have a high potential of growth for generating a positive societal impact.

## 1. Introduction

The explanation of dynamics in sensor research plays a critical role for understanding the evolution of science, technology and human society [1,2,3,4,5] (cf., also how technologies contribute to economic change: [6,7,8,9,10,11,12,13,14]). A sensor is a device, module or subsystem with the goal of detecting events or changes in specific environments and sending the information to other interrelated technological devises, such as a computer processor [3,15,16]. A sensor is also a technology (technology is a complex system, composed of more than one entity or sub-system of technologies and a relationship that holds between each entity and at least one other entity in the system for achieving specific goals, [17]) that interacts with other technologies, having a role either of parasite device (i.e., dependent from other technologies) or host (embodying other technologies) for satisfying needs, achieving goals and solving problems of adopters [18]. For instance, sensors as parasite technology are temperature sensors, proximity sensors, pressure sensors, etc. because they are embodied in other technological systems [19,20]. In general, sensor technologies have multi-mode interactions with other technologies that support a co-evolution of inter-related technological systems and new evolutionary pathways of technological trajectories [17,21,22,23,24,25,26,27,28,29,30,31,32,33,34,35,36]. One main example is smart sensors, which co-evolve through complex interaction with artificial intelligence technologies, Bluetooth technology, medical technologies, cloud computing, etc. [20,22,31,37,38,39,40,41,42,43,44]. New studies show that smart sensors are crucial elements for the Internet of Things [43,45,46,47,48,49,50]. The continuous interactions of sensor technologies with other technologies generate new applications in different fields, such as medicine, environmental science, telematics, the Internet of Things, etc. [17,31,51,52,53].

In this context, the main goal of this article is to analyze sensor research over time to explain the growth and main applications of new sensor technologies for technological and social change. Results here clarify the dynamics of science and new technological trajectories in sensor research that can provide useful information to policymakers for allocating resources and planning scientific and technological development of sensors having positive societal impact. This study is part of a large body of research on the evolution of science and technology that endeavors to explain how research fields and new technologies emerge and evolve in basic and applied sciences [5,10,27,54,55,56,57,58,59,60,61,62].

## 2. Materials and Methods

### 2.1. Study Design for Technological Trajectories

#### 2.1.1. Sources and Sample

The study uses datasets of Scopus over 2021 period [63]. In particular, the window of “Search documents” in Scopus [63] database is used to identify scientific documents (articles and patents) having in title, abstract or keywords the term “sensors”. Scientific products and patents are the basic units for technology and scientific analyses to explain the evolution of science and technology in the field of sensors and to support fruitful policy implications for technological and industrial change [64,65,66]

#### 2.1.2. Measures

▪ Number of articles and all scientific products in “sensors” (conference papers, conference reviews, book chapters, short surveys, letters, etc.), 1955–2020 period.

Data under study here are 1,217,947 document results downloaded in April 2021 [63].

The evolution of sensor research, measured with the number of articles and other scientific products, can show the dynamics of science and technology in this main field.

Additional measure for the analysis of the evolution of sensor technology is:

▪ Number of patents, 1952–2020 period

Patents indicate inventions, and this study analyzes 1,226,074 units over the 1952–2020 period recorded for the field of sensors and its sub-fields.

#### 2.1.3. Specification of the Model and Data Analysis Procedure

The tool “Search documents” in Scopus (2021) provides keywords and time series of documents with the highest frequency of publications in sensor research [63]. After that, sensor technologies with the highest number of documents in the list of keywords have been selected, i.e.,

– wireless sensor networks
– fiber optic sensors
– chemical sensors
– remote sensing
– biosensors
– wearable sensors
– image sensors
– wireless sensors
– optical sensors
– glucose sensors

Each of these keywords are inserted in the window “Search documents” to detect the specific time series for a comparative analysis between sensor technologies, of the list just mentioned, to compute the rate of growth and, consequently, new directions in sensor research. The study applies the model by Sahal for scientific and technology analysis of time series in sensors [67].

Two models are specified as follows.

Firstly,

*Log y_i,t_* = *a* + *b*_1_
*time* + *u_i_*_,_*_t_* (1)

*y_t_* is scientific products *or* patents (dependent or response variable)

*a* is a constant; *b*_1_ is the coefficient of regression.

*log* has base *e* = 2.7182818; *t* = time; *u* = error term in equation.

The parameters *a* and *b* in model [1] are unknown and estimated using the data of sample in the Ordinary Least Squares (OLS) method.

Secondly, if we consider the ratio:

$$\delta_{i,t}=\frac{number\;of\;publications\;\left(or\;patents\right)\;in\;the\;subfield\;i\;of\;sensors\;at\;t}{Total\;number\;of\;publications\;\left(or\;patents\;\right)\;at\;t}$$

The specification of the model is:

*Log δ_i_*_,_*_t_* = *a’* + *b*_1′_
*time + ε_i,t_* (2)

The equation [2] also has *a’* = constant; *b*_1′_ = coefficient of regression (*a’* and *b’* are the parameters to be estimated); *t* = time; *ε* = error term in equation.

This relationship [2] here is also investigated with OLS method for estimating the unknown parameters with a regression model [68].

Statistical analyses are performed with the IBM SPSS Statistics 26 ^®^.

### 2.2. Technological Analysis within Research Fields of Sensors to Detect Technological Characteristics and Applications 

#### 2.2.1. Research Settings

The methodology here has the purpose to investigate the structure of emerging research fields in sensor technology, detected with previous statistical analysis by the highest coefficients of regression in estimated relationships based on publication and patent data (Equations (1) and (2)); high magnitude of coefficients of regression is a proxy of high evolutionary growth of technological trajectories in sensor research over time. Emerging research fields under study here, having the highest coefficients of regression, are given by:

**□**  Wireless sensor networks. A wireless sensor network is a group of objects that transfer the gathered data through multiple nodes and wireless infrastructure to cooperatively sense and control the environment [69]. These devices are positioned in large numbers, so they need the ability to assist each other to transfer data back to a centralized collection point [70].
**□**  Wearable sensors. Wearable sensors are integrated into wearable objects attached to the body for health monitoring or relevant data collection. They have diagnostic and monitoring applications, including physiological and biochemical sensing and motion sensing [71]. Wearable sensor adaptation has involved miniaturizing sensing technologies, making them comfortable and flexible, and developing software that increases the value of measured data [72].
**□**  Biosensors. A biosensor is an analytical device that measures biological or chemical sensing elements and reactions. Biosensors are generally employed for monitoring pollutants, health parameters, biomarkers, etc. [73]. They restrain biology’s great sensitivity and specificity in intersection with physicochemical transducers to provide detailed and bioanalytical measurements with easy-to-use and straightforward formats [74].

This section applies Natural Language Processing (NLP) to demonstrate common research themes in emerging subfields of sensors just mentioned (i.e., wireless sensor networks, wearable sensors and biosensors). In the document type section of the Scopus dataset [63], the data of conference paper, article, conference review and review have been collected. Among statistical algorithms, topic modelling as a text-mining tool can help to discover and organize latent topics. This modelling allows us to create an extensive semantic structure of a text body through various disciplines’ correlations [75]. We implemented the Latent Dirichlet Allocation (LDA) as an unsupervised approach for topic modelling (i.e., machine learning-LDA) that attract popularity in textual data processing because of its ability to reduce the bias and increase the accuracy for literature investigation [76]. Moreover, we used java implementation of this model with the name MALLET [77]. In this study, we used the Python programming language for building a topic model. The methodology has been accomplished in three steps: (1) data gathering and text pre-processing, (2) topic construction and (3) investigation, which are explained in more details.

#### 2.2.2. Sources of Data, Sample and Measures of Computational Analyses

This study, as said, uses data from Scopus [63]. According to search procedures, we have obtained:

○ 1989 publications in wireless sensor networks published from 1989 to 2020, including keywords in articles’ keywords, abstract and title.
○ 71,780 articles in wearable sensors published from 1998 to 2020.
○ 66,996 documents in biosensors published from 1970 to 2020.

After an initial review of these articles, the abstracts were used to input the LDA technique to explore topics under study. Measures are similar and described in the previous section.

#### 2.2.3. Topic Modelling and Data Analysis Procedure

1. Step 1: data gathering and text pre-processing

This study employed data from the Scopus (2021) database [63]. For collecting the related documents, we used the search string TITLE-ABS-KEY (“wearable sensor”) for wearable sensor papers, TITLE-ABS-KEY (“Biosensor”) for Biosensor papers, and TITLE-ABS-KEY (“Wireless sensor network”) for Wireless sensor network documents. All publications were collected until 2020, and for increasing the accuracy of data, this study limited the records to conference papers, article, conference reviews and reviews in English.

Secondly, for textual data pre-processing, we conducted a topic modelling analysis using Python 3.7.7 version programming language to first concatenating all abstracts of publications and then concatenating them into one string set for each field. We created a corpus of the respective field documents by which the model learns the ‘topics’. The data are pre-processed prior to the topic modelling using GenSim library [78] to convert each publication’s abstract into a bag-of-words representation. We consider each word as a token and then eliminated words in a stopword list provided in the MALLET software [77]. Then, words with a low frequency, fewer than three characters were removed. We exerted the Tokenization technique by splitting the text into a set of words, doing punctuation removal and adjusting the terms with higher cases into lowercase. Aside from those processes, we implemented lemmatization to assimilate all the verbs in various tenses to present tenses and modified them to the first person. In the end, we removed all terms that appear fewer than ten times across all documents, or that appear in more than 70 percent of records.

2. Step 2: topic construction

**Figure 1:**
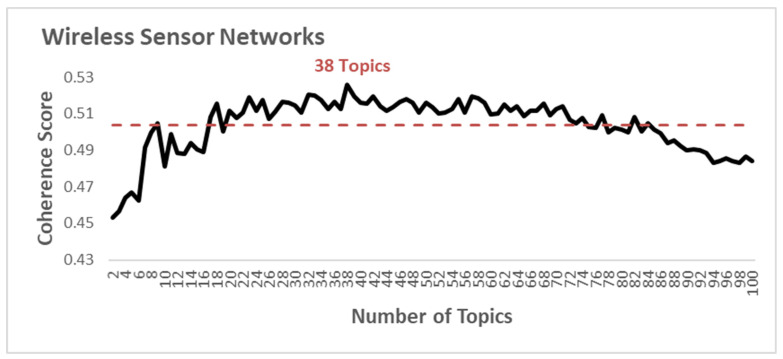

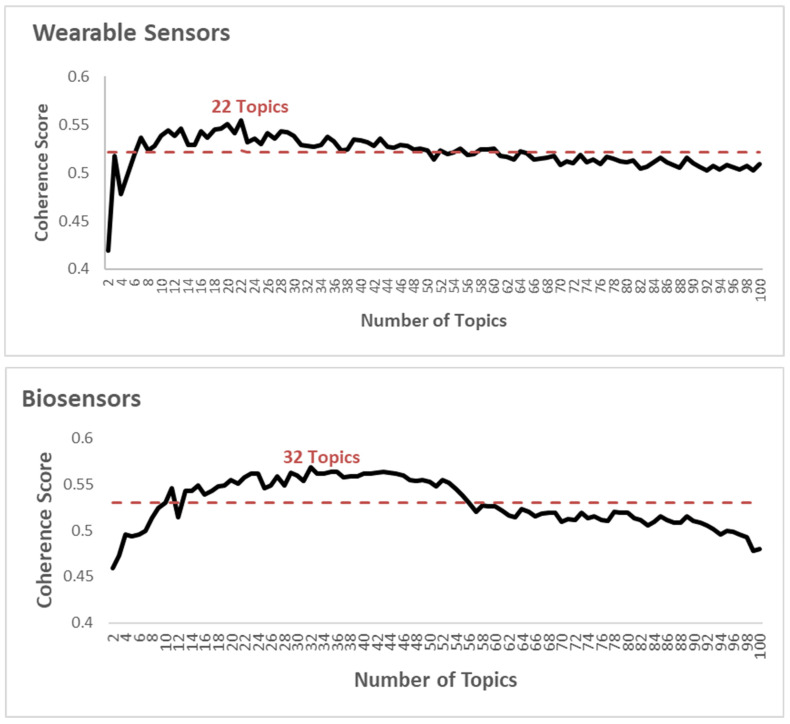
Topic coherence score with a different number of topics in wearable sensor, biosensor and wireless network sensor with the sliding window size of 100.

We can assume a topic as a probability distribution over a term. Those vocabularies with a high probability of occurrence in the same topic are more likely to appear frequently in the same documents simultaneously. For constructing the topic, we started training the model using MALLET, a Java-based package used for statistical NLP developed by McCullum [77] to build a Latent Dirichlet Allocation model (LDA). This model requires a fixed number of topics that is not specified accurately for a corpus. Accordingly, we chose an optimal number of topics for implementing the topic modelling technique following the study by Mifrah and Benlahmar [79]. In this respect, we calculated the topic coherence score for each number of topics to identify the most efficient one. We used the C_v coherence measure to retrieve co-occurrence counts of respective word sets based on the sliding window size. We calculated the normalized pointwise mutual information (NPMI) for every top word to extract a set of vectors for each top word. Afterwards, we measured the similarity between the top words sum vector and each top word vector in one-set segmentation. We utilized cosine similarity to calculate the coherence score based on an arithmetic mean of all similarities [79]. We calculated the coherence of a couple of models through different numbers of topics according to the approach of Röder to identify the best number of topics for our model applied in the present study [80]. Figure 1 demonstrates the coherence score of the model through the different numbers of topics. For wearable sensors, results show that the highest coherence value (i.e., 0.5546) occurs in topic number 22; for biosensors, the highest coherence value (i.e., 0.5687) occurs in topic number 32; and for wireless sensor networks, the greatest coherence value (i.e., 0.5260) stands for topic number 38.

3. Step 3: Investigation

In this step, the study here investigated topics of the emerging research fields in sensor technology described before: wireless sensor networks; wearable sensors and biosensors. This section presents topic modeling findings using a word-cloud demonstration in which the size of each word in a specific topic is done according to its frequency in that topic. Afterward, we classified all topics of each field into two categories: technological characteristics and applications. In the second part of the results, trend analysis was conducted separately to demonstrate their evolutionary growth based on the popularity of topics over time. Evolutionary growth of topics within each research field under study (wireless sensor networks, wearable sensors, and biosensors) has been categorized in Positive Evolutionary Growth, Stable Evolutionary Growth and Negative Evolutionary Growth to assess the topic trend analysis for the classification of each emerging subfields of the sensor. In particular,

- Positive Evolutionary Growth indicates that the topic popularity has been increasing, and the occurrence frequency of the topic words has been elevating.
- Stable Evolutionary Growth indicates that the topic popularity has been fluctuating and does not follow a trend of growth or decline. It means that the occurrence frequency of the words in topic has stable evolution.
- Negative Evolutionary Growth indicates that the topic popularity has been decreasing, and the occurrence frequency of the topic words has faced reduction.

## 3. Results and Discussion

### 3.1. Growth of Research Fields in Sensors

The parametric estimates of models (1–2), based on scientific production, are presented in Table 1. In many cases, the significance of the coefficients of regression and the explanatory power of equations has *p*-value < 0.001. The coefficient of R^2^ has high values and in general the models explain more than 80% variance in the data.

Table 2 shows the parametric estimates of models (1–2) based on patents. Table 2 also reveals that in most cases, the significance of the coefficients of regression and the explanatory power of equations has *p*-value < 0.001, except model (2) for remote sensing. The R^2^ has also here high values and in a majority of cases the models explain more than 70% variance in the data.

**Figure 2:**
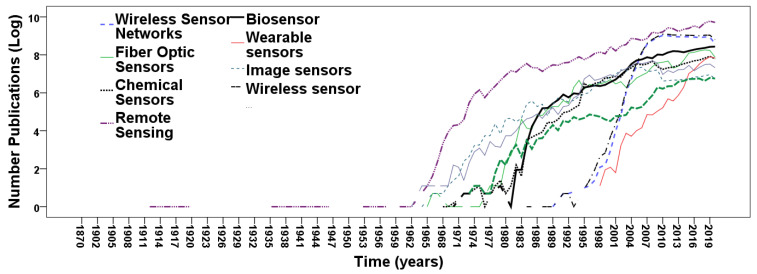
Trends of research fields in sensors using scientific production (*log* scale).

**Figure 3:**
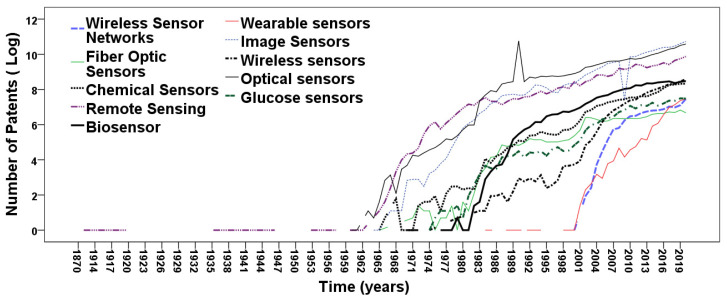
Technological trajectories of sensor using patents (*log* scale).

Table 3 shows the coefficients of regression of models calculated in Table 1 and Table 2, and suggests that the emerging research fields in sensors are (trends are displayed in Figure 2 and Figure 3):

- wireless sensor networks
- wearable sensors
- biosensors

Results also suggest that wireless sensors, a restriction of wireless sensor networks, have a high evolutionary growth in the field of sensor technology. All these research fields are the younger ones among research fields in sensors. This result is consistent with the studies by Coccia [10,58] that higher growth rates of scientific production are in new research fields rather than old ones.

The next section shows results to clarify structure of sensor research and to detect critical technological characteristics and applications for progress in science and society.

### 3.2. Structure, Characteristics, and Applications of Critical Research Fields in Sensors

The results of topic modelling analysis demonstrate the top 15 high-frequency terms in each topic. These topics contain the words reflecting the content and terms of documents with the highest score. The topics are related to significant issues in each growing subfield in sensor technology. We illustrated 38 topics in wireless sensor networks, 22 topics in wearable sensors and 32 topics in biosensors through a word-cloud analysis; the size of each word indicates comparatively the frequency weight of a term in a specific case. The larger the word, the higher the frequency stands in the parent topic. Accordingly, this visualization can reflect the brief information of each topic and partially explains the included documents. Ultimately, this study analyzes and explores the evolution of these topics over time. Topic modeling analysis can also demonstrate the increasing or decreasing popularity of topics in sensor research, which can better explain how a field of research has been changing over time. We normalized the proportion of each topic per year and obtained the annual trends.

#### 3.2.1. Wireless Sensor Networks

**Figure 4:**
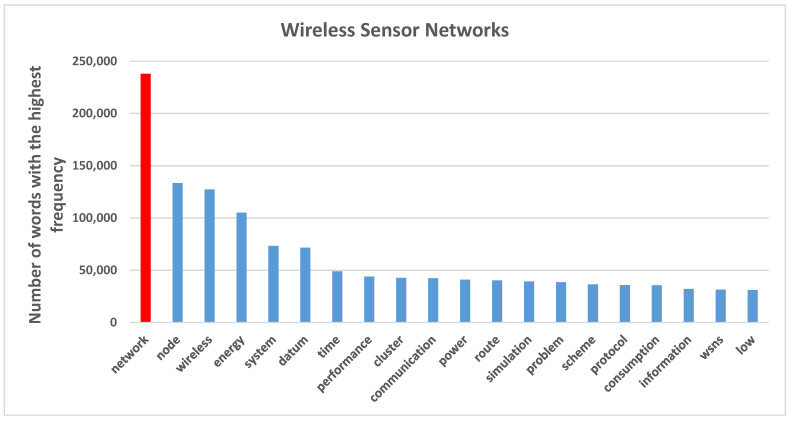
The highest frequent words in documents of wireless sensor networks.

Figure 4 shows the 20 most frequent words that appeared in publications on wireless sensor networks. Our results show that the terms “network”, “node”, “wireless” and “energy” have been used more than 100,000 times across the corpus. Each word, according to its similarity regarding the co-occurrence, leads to topics creation.

**Figure 5:**
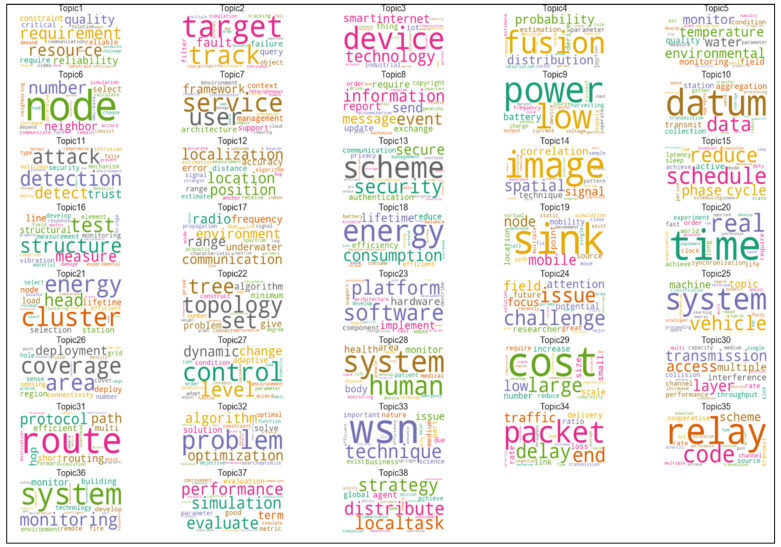
Word-Cloud in documents of wireless sensor networks.

Figure 5 shows the topic’s classification of the wireless sensor network. The largest words of each class represent the content of the topic documents. Figure 5 of Word-Cloud analysis suggests information about technological characteristics and applications of wireless network sensors.

Main technological characteristics of wireless sensor networks are (from Figure 5):

- Internet of Things
- network optimization
- data security
- monitoring system
- optimization
- technical infrastructure

Instead, the main application characteristics of wireless sensor networks are (from Figure 5):

- environmental monitoring
- communication systems
- energy
- smart vehicles
- control systems
- healthcare

Table 4 shows the evolutionary growth of topics in wireless network sensors. From this classification, it can be concluded that the studies of smart sensors associated with the Internet of Things are growing; the studies of environmental monitoring and health care evolutionary level are also increasing over time.

This study reveals that networking of sensor systems is growing over time [48,52,81,82,83]. Results here also suggest that wireless sensor networks have a higher rate of evolution likely because of the interaction with specific technologies, such as Internet of Things, data security and monitoring systems. In the context of technological applications, these sensors have a growing application in environmental monitoring and healthcare sector [84,85,86,87]. A critical aspect in these sensors is the maintenance, and many sensors’ wireless systems are powered with batteries or self-powering technology. Ultra-low-power sensors are a desirable option because they can reduce the need of regular battery changes and support a higher technological sustainability in environment [88]. Finally, technology of wireless network sensors has the advantage of easy upgrades of new technological characteristics; consequently, the technological system can be more efficient from a technological and economic point of view [51,89].

#### 3.2.2. Wearable Sensors

**Figure 6:**
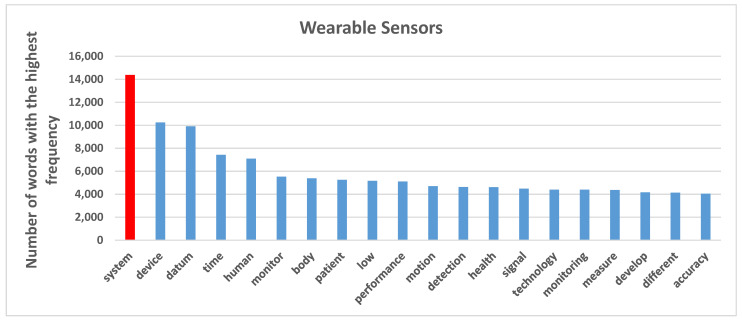
The highest frequent words in documents of wearable sensors.

Figure 6 shows the top 20 words with the highest frequency in publications of wearable sensor. These findings reveal that the terms “system”, “device”, “datum”, “time” and “human” have appeared more than 6000 times across the corpus.

**Figure 7:**
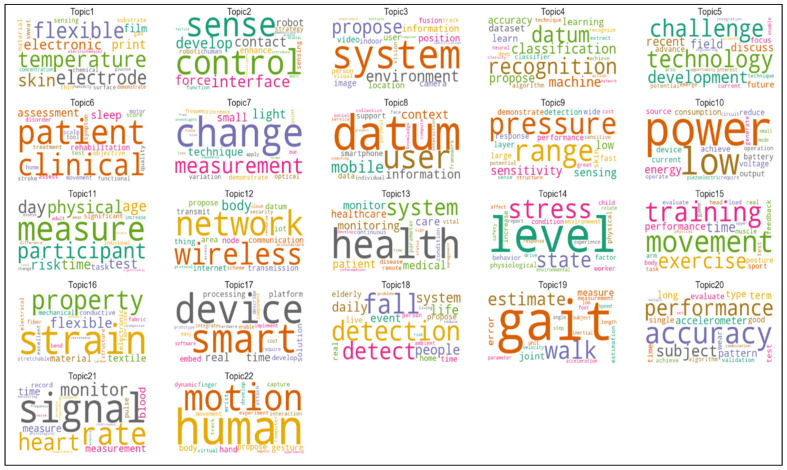
Word-Cloud in documents of wearable sensors.

Figure 7 illustrates 22 topics of wearable sensor documents by interpreting the most important words with the highest frequency of occurrence. Each category represents publications. A more comprehensive insight from this analysis is the categorizations of topics according to technological characteristics and applications of sensors.

Critical technological characteristics of wearable sensors are (Figure 7):

- sensor particles
- machine learning
- monitoring
- biosensing technologies
- pressure sensing
- detection technologies
- sensor network

Instead, the application characteristics of wearable sensors are (Figure 7):

- energy and power
- physical activities
- medical science
- psychology

Table 5 shows the positive popularity rate of wearable sensor technologies over time, such as sensor particles, machine learning and pressure sensing. The growing application topics are mainly physical activities and body motion measuring.

Results also show that wearable sensors can be biosensors connected to the body for assessing different biological elements; therefore, the healthcare system is one of the essential applications [52,90,91]. In fact, the embedding of wearable sensor systems in health treatment procedures reduces the cost of hospitals’ daily expenditures. These facilities enable doctors to remotely monitor patients’ health conditions, reducing extended stays in hospitals and maintenance costs of these structures [92,93,94]. Results also suggest that pressure sensing is a main technological characteristic of wearable sensors [95,96]. However, wearable sensors are still facing several challenges. One of the most problematic issues is related to the adaptability of sensors to the body to be comfortable in body-worn devices. This study confirms that flexible, stretching, and soft technologies are growing to enable wearable devices to be more usable in daily life activities [97]. Such technologies and human motion sensing analysis studies are rising because of the importance of disabled people’s living conditions enhancement [98,99]. In this context, results here suggest that future developments are directed to improve material flexibility, softness and comfort of the wearable technologies (e.g., in artificial legs and hands devices) to be used properly in many patients [100].

#### 3.2.3. Biosensors

**Figure 8:**
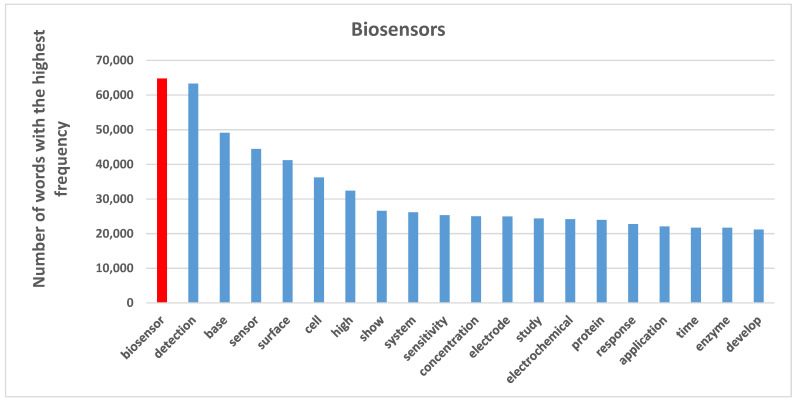
The highest frequent words in documents of biosensors.

Biosensors have shown great potential in many areas, such as clinical diagnostics, food analysis, bio process and environmental monitoring. Biosensors are, depending on the method of signal transduction—optical, mass, electrochemical, magnetic, micromechanical and thermal sensors. Moreover, biosensors can use a combination of biological receptor compounds (antibody, enzyme, nucleic acid, etc.) and the physical or physic–chemical transducer directing, in most cases, “real–time” observation of a specific biological event (e.g., antibody–antigen interaction). Figure 8 shows the 20 words with the highest occurrence in biosensors. Our findings reveal that the terms “biosensor”, “detection”, “base”, “sensor”, “surface”, “cell” and “high” have the highest frequency, appearing more than 30,000 times in the corpus. These high-frequency words’ similarity regarding their co-occurrence matrix have been considered in topic creations.

**Figure 9:**
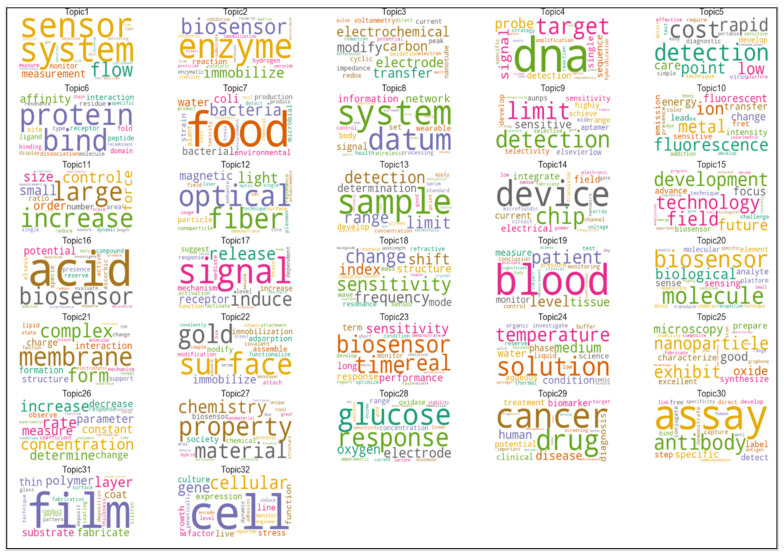
Cloud words in documents of biosensors.

Figure 9 illustrates 32 topics of biosensor documents. The largest words with the highest occurrence frequency in each category partially represent the content of documents. Topics of biosensors, visualized in Figure 9, can be also categorized in technological characteristics and applications to see technology-oriented and application-oriented aspects of this research and technological field.

Main technological characteristics of biosensors are (Figure 9):

- measurement sensors
- electrochemical sensors
- detection technologies
- network sensors
- optical sensors
- nanotechnology
- glucose sensors

Instead, main application characteristics of biosensors are (Figure 9):

- genetic
- DNA sequence
- vital sign measurement
- cancer detection
- patient monitoring

Table 6 shows that material science, nanotechnology and detection processes have been growing in sensor research to expand the technological aspects of biosensors. Moreover, this analysis demonstrates that the glucose sensors topic faced a considerable reduction in its popularity.

The results here also demonstrate that biosensor studies are growing over time, especially in topics associated with detecting and monitoring applications in medical systems [101]. In addition, nano sensor technologies have started to interacting with other technologies, improving the efficiency of biosensor performance to reduce human error in disease detection and the cost of human resource in the healthcare industry [46,102]. One of the aspects that supports the growth of biosensors is the emergence of biochemical sensors containing active materials in their chemical structures to assess biological or chemical reactions by generation of signals to identify and measure the concentration of an analyte in the reaction. These technologies have been utilized mainly for detection purposes, including biomarker detection for blood, glucose level, food mass, anti-body, genetic aspects, etc. [103,104,105]. We should also consider that the Coronavirus Disease 2019 (COVID-19) pandemic crisis has changed health systems and supported these technologies requiring a rapid detection by immunosensor of patients and their remote monitoring [106]. In fact, one of the fundamental problems in pandemic control is the insufficient capacity of hospitals to hospitalize, at the same time, infected individuals with serious symptoms of COVID-19 [18,107,108,109]. Hence, biosensor technologies, associated with other sensors, enable doctors to monitor and treat patients remotely, in their house, instead of in the hospital, helping the healthcare management of patients, reducing costs and the negative effects of this novel coronavirus in society. Overall, then, the biosensor is gaining momentum to detect and monitor remotely patients affected of the COVID-19, patients with other disorders and/or post-surgical patients to reduce the total cost of healthcare and improve the efficiency of hospitals [110,111,112,113,114,115].

## 4. Conclusions, Limitations and Prospects

**Figure 10:**
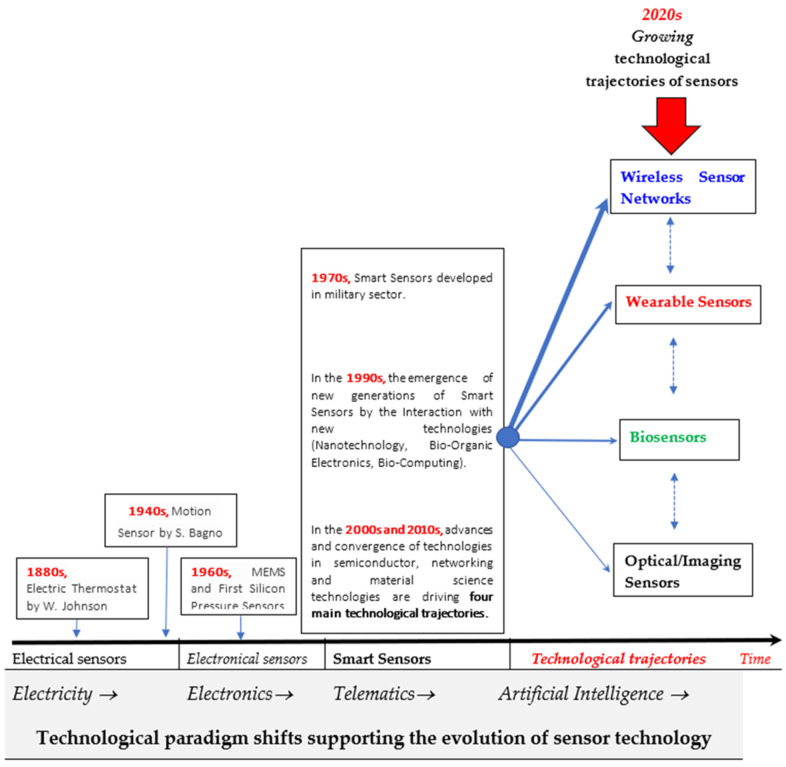
Macro evolution of sensor technology from electrical, (micro) electronic and smart sensors with scientific fields and technologies having high growth for pervasive and innovative development in industrial sectors. *Note*: A sensor is a device that detects changes in quantities. A greater (*smaller*) thickness of arrows indicates a higher (*lower*) intensity of scientific and technological growth of sensor technological trajectory, considering the coefficients of regression in Table 3.

This study shows that in sensor research, high growth rates are associated with research fields of wireless sensor networks, wearable sensors and biosensors, supporting new directions for scientific and technological development in society (Figure 10). The general evolution of sensor technology is driven by technological paradigm shifts and new technological regimes that have supported the progress from electrical sensors (with the technological revolution of electricity), to electronical sensors (with the technological revolution of electronics and microelectronics), to smart sensors (with the technological revolution of telematics) and now towards new technological frontiers with the technological revolution of artificial intelligence, cloud computing, internet of things, etc. (Figure 10).

This study reveals that technological development of sensors is due to evolutionary pathways based on interactions of sensors with other technological systems, such as information and communication technologies, artificial intelligence, Internet of Things, etc. [33,34,36,43,45,46,49,50] (cf., also [28,47,48,54,116,117,118,119,120,121,122]). Results suggest that sensors have, as parasite technologies (i.e., depending on other technologies; [17]), a wide spectrum of applications in medicine, environmental pollution, aircraft and automotive industries [123,124,125,126,127]. Moreover, the success of smart sensors is associated with the integration of the Internet of Things, through which it is possible to connect devices and exchange information among people, systems, objects and many other devices [128]. Historically, research and development (R&D) efforts in sensor technology have been funded as an adjunct to large application programs that required sensors [16]. Now, selected R&D investments support the development of new and improved sensors with effective research planning processes directed to users for specific applications [129]. The description here of new technological directions and characteristics of sensors, having improved performance capabilities and applications in different settings, can help policymakers to enhance the allocation of R&D investments in private and public research organizations for scientific and technological development, and technology transfer of new sensors in society [56,64,130,131,132,133,134,135,136].

This study also shows that sensor research is a vast research field in continuous evolution because of recent advances in information and communication technologies, artificial intelligence, nanoscience, human-computer interaction, cloud computing, etc. that enable intensive interactions of sensor technology with other disciplines and technologies. Overall, then, this study maintains that growing fields in sensor research are given by wireless sensor networks, wearable sensors and biosensors with new applications in environmental, sustainability and health sciences. However, these conclusions here are of course tentative. We know that other things are not equal in the science dynamics of sensor research and there is need for much more detailed examinations to explain other directions in the design, implementation and evaluation of interactive technology of sensors in society. The future development of this study is directed to reinforce this study with additional data to support the proposed empirical results here and extend the investigation on scientific ecosystem of sensors over time in order to clarify the advances of intelligent sensors in the presence of computing interactions, smart environments, human-machine interactions and/or virtual and augmented reality.

## Figures and Tables

**Table 1:** Estimated relationships of scientific production in research fields of sensors as a function of time.

Dependent Variable: Scientific Products Concerning Research Fields in Sensors					
Research Fields	Coefficient *b*_1_, and *b’*_1_	Constant a	F-Test	R^2^	N, Period
Wireless Sensor Networks, *Log y_i,t_*	0.35 ***	−695.45 ***	141.64 ***	0.85	N = 27 (1989–2020)
*Log δ_i,t_*	0.24 ***	−490.02 ***	140.46 ***	0.82	
Fiber Optic Sensor, *Log y_i,t_*	0.17 ***	−324.33 ***	432.74 ***	0.90	N = 51 (1965–2020)
*Log δ_i,t_*	0.05 ***	−100.24 ***	38.17 ***	0.43	
Chemical Sensor, *Log y_i,t_*	0.17 ***	−339.06 ***	345.42 ***	0.89	N = 46 (1968–2020)
*Log δ_i,t_*	0.06 ***	−130.48 ***	54.10 ***	0.55	
Remote sensing, *Log y_i,t_*	0.13 ***	−241.34 ***	304.89 ***	0.84	N = 60 (1956–2020)
*Log δ_i,t_*	−0.002	1.96	0.18	0.003	
Biosensors, *Log y_i,t_*	0.18 ***	−343.25 ***	255.47 ***	0.86	N = 43 (1970–2020)
*Log δ_i,t_*	0.07 ***	−137.53 ***	47.34 ***	0.53	
Wearable sensors, *Log y_i,t_*	0.30 ***	−598.27 ***	766.26 ***	0.97	N = 22 (1998–2020)
*Log δ_i,t_*	0.21 ***	−421.51 ***	406.37 ***	0.95	
Image sensors, *Log y_i,t_*	0.12 ***	−223.08 ***	236.66 ***	0.81	N = 55 (1964–2020)
*Log δ_i,t_*	−0.004	3.95	0.48	0.009	
Wireless sensor, *Log y_i,t_*	0.34 ***	−679.77 ***	221.60 ***	0.88	N = 30 (1984–2020)
*Log δ_i,t_*	0.24 ***	−490.02 ***	140.46 ***	0.83	
Optical sensors, *Log y_i,t_*	0.13 ***	−255.65 ***	562.65 ***	0.91	N = 56 (1962–2020)
*Log δ_i,t_*	0.008 *	−20.44 *	3.64 *	0.06	
Glucose sensors, *Log y_i,t_*	0.12 ***	−243.19 ***	584.69 ***	0.93	N = 47 (1973–2020)
*Log δ_i,t_*	0.02 ***	−43.14 ***	15.72 ***	0.26	

*Note*: Explanatory variable is time in years. N is the number of observations from the specified period (the first year indicates the first paper recorded, the second year is 2020 because 2021 is still ongoing). *** significant at 1‰; * significant at 5%. F is the ratio of the variance explained by the model to the unexplained variance; R^2^ is the coefficient of determination adj.

**Table 2:** Estimated relationships of patents in research fields of sensors as a function of time.

Dependent Variable: Patents Concerning Fields of Research in Sensors					
Research Fields	Coefficient *b*_1_, and *b’*_1_	Constant a	F-Test	R^2^	N, Period
Wireless Sensor Networks, *Log py_i,t_*	0.30 ***	−591.58 ***	60.02 ***	0.77	N = 19 (2000–2020)
*Log pδ_i,t_*	0.21 ***	−430.12 ***	41.72 ***	0.70	
Fiber Optic Sensor, *Log py_i,t_*	0.14 ***	−272.48 ***	291.16 ***	0.86	N = 50 (1971–2020)
*Log pδ_i,t_*	0.03 ***	−59.57 ***	12.64 ***	0.21	
Chemical Sensor, *Log py_i,t_*	0.16 ***	−314.77 ***	1293.12 ***	0.96	N = 54 (1965–2020)
*Log pδ_i,t_*	0.04 ***	−92.14 ***	92.52 ***	0.64	
Remote sensing, *Log py_i,t_*	0.13 ***	−240.97 ***	304.30 ***	0.84	N = 60 (1956–2020)
*Log pδ_i,t_*	−0.002	2.50	0.24	0.004	
Biosensors, *Log py_i,t_*	0.20 ***	−383.42 ***	255.38 ***	0.86	N = 43 (1978–2020)
*Log pδ_i,t_*	0.09 ***	−181.04 ***	59.81 ***	0.59	
Wearable sensors, *Log py_i,t_*	0.25 ***	−492.18 ***	283.88 ***	0.93	N = 24 (1984–2020)
*Log pδ_i,t_*	0.15 ***	−304.52 ***	98.78 ***	0.81	
Image sensors, *Log py_i,t_*	0.18 ***	−340.36 ***	438.04 ***	0.89	N = 55 (1964–2020)
*Log pδ_i,t_*	0.06	−112.64	68.68	0.56	
Wireless sensor, *Log py_i,t_*	0.22 ***	−425.83 ***	837.44 ***	0.96	N = 39 (1974–2020)
*Log pδ_i,t_*	0.11 ***	−232.03 ***	268.89 ***	0.88	
Optical sensors, *Log py_i,t_*	0.16 ***	−313.61 ***	372.72 ***	0.87	N = 59 (1960–2020)
*Log pδ_i,t_*	0.03 ***	−65.57 ***	29.65 ***	0.34	
Glucose sensors, *Log py_i,t_*	0.15 ***	−300.56 ***	663.05 ***	0.94	N = 46 (1974–2020)
*Log pδ_i,t_*	0.05 ***	−100.51 ***	84.23 ***	0.65	

*Note*: Explanatory variable is *time in years*. *N* is the number of observations from the specified period (the first year indicates the first paper recorded, the second year is 2020 because 2021 is still ongoing). *** significant at 1‰. *F* is the ratio of the variance explained by the model to the unexplained variance; R^2^ is the coefficient of determination adj.

**Table 3:** Evolutionary growth of scientific fields in sensor technology considering the coefficients of regression based on number of publications and patents over time, and their scientific age from the first scientific products published to the year 2020.

Research Fields	Coefficient of Regression (Publications)	Age	Research Fields	Coefficient of Regression (Patents)	Age
Wireless Sensor Networks	0.35	31	Wireless Sensor Networks	0.30	31
Wireless sensor	0.34	36	Wearable sensors	0.25	22
Wearable sensors	0.30	22	Wireless sensor	0.22	36
Biosensors	0.18	50	Biosensors	0.20	50
Fiber Optic Sensor	0.17	55	Image sensors	0.18	56
Chemical Sensor	0.17	52	Chemical Sensor	0.16	52
Remote sensing	0.13	64	Optical sensors	0.16	58
Optical sensors	0.13	58	Glucose sensors	0.15	47
Image sensors	0.12	56	Fiber Optic Sensor	0.14	55
Glucose sensors	0.12	47	Remote sensing	0.13	64

**Table 4:** Dynamics of trends in wireless sensor networks using trend analysis.

	Number of Topics
Positive Evolutionary Growth	3 (smart device, internet of things, etc.), 5 (environmental, water, temperature, monitor, etc.), 24 (future, potential, challenge, etc.), 28 (system, human, health, etc.), 33 (WSN, technique, business, etc.)
Stable Evolutionary Growth	1 (resource, reliability, etc.), 2 (target, track, etc.), 4 (fusion, distribution, etc.), 6 (node, neighbor, etc.), 7 (service framework, architecture, etc.), 8 (information, report, etc.), 9 (power, low, battery, etc.), 10 (datum, aggregation, transmit, etc.),11 (attack, detection, trust, etc.), 12 (localization, position, location, etc.), 13 (scheme, security, communication, etc.), 14 (image, signal, etc.), 15 (schedule, phase cycle, etc.), 16 (structure, test, measure, etc.), 17 (radio, frequency, communication, etc.), 18 (energy, consumption, etc.), 19 (sink, mobility, node, etc.), 20 (real, time, etc.), 21 (energy, head, cluster, etc.), 23 (platform, software, hardware, etc.),25 (system, vehicle, machine, etc.), 26 (deployment, coverage, area, etc.), 27 (control dynamic, level, etc.), 29 (human, system, body, etc.), 30 (transmission, access, layer, etc.), 31 (protocol, route, path, etc.), 32 (algorithm, problem, optimization, etc.), 34 (traffic, packet, delay, etc.), 35 (relay, code, scheme, etc.), 36 (monitoring, system, etc.), 37 (performance, evolution, simulation, etc.), 38 (distribution, local task, strategy, etc.)
Negative Evolutionary Growth	22 (topology, algorithm, tree, etc.)

**Table 5:** Dynamics of trends in wearable sensors using trend analysis.

	Number of Topics
Positive Evolutionary Growth	1 (electronic, electrode, temperature, etc.), 4 (datum, recognition, machine learning, etc.), 9 (pressure sensing, range, etc.), 11 (measure, physical, risk, etc.), 16 (strain, flexible, material, etc.)
Stable Evolutionary Growth	2 (sense, control, robot, etc.), 5 (future, technology, challenge, etc.), 6 (patient, clinical, etc.), 7 (change, measurement, etc.), 14 (stress, level, etc.), 15 (training, movement, exercise, etc.), 19 (estimate, gait, walk, etc.), 20 (performance, accuracy, accelerometer, etc.), 21 (signal, heart rate, etc.), 22 (motion, human, etc.)
Negative Evolutionary Growth	3 (environment, system, position), 8 (datum, mobile, smartphone, etc.), 10 (power, energy, battery), 12 (wireless, network, body, etc.), 13 (healthcare, system, monitoring, etc.), 17 (smart, device, real-time, etc.), 18 (detection, daily, system)

**Table 6:** Dynamics of trends in biosensors using trend analysis.

	Number of Topics
Positive Evolutionary Growth	9 (detection, sensitivity, etc.), 25 (nanoparticle, microscopy, etc.), 27 (chemistry, material, etc.)
Stable Evolutionary Growth	1 (sensor system, fellow, measurement, etc.), 3 (electrochemical, electrode, carbon, etc.), 4 (DNA, signal, etc.), 5 (detection, point, etc.), 6 (protein, bind, affinity, etc.), 7 (food, bacterial, environment, etc.), 8 (system, datum, etc.), 10 (metal, fluorescence, etc.),11 (size control, etc.), 12 (optical fiber, magnetic, etc.), 13 (detection, sample, etc.), 14 (device, chip, etc.), 15 (technology, development, future, etc.), 16 (acid, biosensor, etc.), 17 (signal, release, etc.), 18 (sensitivity, frequency, etc.), 19 (patient, blood, etc.) 20 (biosensor, molecule, biological, etc.),21 (complex, membrane, etc.), 22 (gold, surface, etc.), 23 (biosensor, real-time, sensitivity, etc.), 24 (temperature, solution, etc.), 29 (cancer, drug, biomarker, etc.), 30 (assay, anti-body, etc.), 31 (film, layer, polymer, etc.), 32 (cell, cellular, gene, etc.)
Negative Evolutionary Growth	2 (biosensor, enzyme, immobilize, etc.), 26 (measure, parameter, concentration, etc.), 28 (glucose, response, electrode, etc.)

## Data Availability

*E*-mail to: mario.coccia@cnr.it (Mario Coccia).
